# Increased Postural Demand Is Associated With Greater Cognitive Workload in Healthy Young Adults: A Pupillometry Study

**DOI:** 10.3389/fnhum.2018.00288

**Published:** 2018-07-18

**Authors:** Melike Kahya, Tyler A. Wood, Jacob J. Sosnoff, Hannes Devos

**Affiliations:** ^1^Department of Physical Therapy and Rehabilitation Science, School of Health Professions, University of Kansas Medical Center, Kansas City, KS, United States; ^2^Department of Kinesiology and Community Health, University of Illinois at Urbana–Champaign, Urbana, IL, United States

**Keywords:** posture, dual-tasking, cognitive-motor interference, cognitive workload, pupillometry, healthy young

## Abstract

**Introduction:** Balance tasks require cognitive resources to ensure postural stability. Pupillometry has been used to quantify cognitive workload of various cognitive tasks, but has not been studied in postural control. The current investigation utilized pupillometry to quantify the cognitive workload of postural control in healthy young adults. We hypothesized that cognitive workload, indexed by pupil size, will increase with challenging postural control conditions including visual occlusion and cognitive dual tasking.

**Methods:** Twenty-one young healthy adults (mean ± standard error of the mean), (age = 23.2 ± 0.49 years; 12 females) were recruited for this study. Participants completed four tasks: (1) standing with eyes open; (2) standing with eyes occluded (3) standing with eyes open while performing an auditory Stroop task; and (4) standing with eyes occluded while performing an auditory Stroop task. Participants wore eye tracking glasses while standing on a force platform. The eye tracking glasses recorded changes in pupil size that in turn were converted into the Index of Cognitive Activity (ICA). ICA values were averaged for each eye and condition. A two-way Analysis of Variance with *post-hoc* Sidak correction for pairwise comparisons was run to examine the effect of visual occlusion and dual tasking on ICA values as well on Center of Pressure (CoP) sway velocity in anterior–posterior (AP) and medio-lateral (ML) directions. A Pearson’s correlation coefficient was utilized to determine the relationship between ICA values and CoP sway velocity.

**Results:** Significant within-condition effect was observed with visual occlusion for the right eye ICA values (*p* = 0.008). Right eye ICA increased from eyes open to eyes occluded conditions (*p* = 0.008). In addition, a significant inverse correlation was observed between right eye ICA values and CoP sway velocity in the ML direction across all the conditions (*r* = -0.25, *p* = 0.02).

**Conclusion:** This study demonstrated support for increased cognitive workload, measured by pupillometry, as a result of changes in postural control in healthy young adults. Further research is warranted to investigate the clinical application of pupillometry in balance assessment.

## Introduction

Balance tasks involve the use of many different motor and sensory systems to integrate environmental stimuli in order to maintain postural stability (Pollack et al., 2000; Mancini and Horak, 2010; Muir et al., 2012; Aartolahti et al., 2013). The integration and coordination of the multiple systems to complete a movement require cognitive resources (Muir et al., 2012; Aartolahti et al., 2013; Muir-Hunter and Wittwer, 2016). Increased motor task difficulty will exert greater cognitive resources (Muir et al., 2012; Aartolahti et al., 2013; Muir-Hunter and Wittwer, 2016). Dual task interference has been used to examine deteriorations in motor performance when the demand of a combined cognitive and motor task exceeds the available cognitive resources (Woollacott and Shumway-Cook, 2002; Siu et al., 2008; Al-Yahya et al., 2011; Plummer-D’Amato et al., 2012; Plummer et al., 2013). In healthy young adults, it has been shown that postural control requires a small amount of cognitive resources (Woollacott and Shumway-Cook, 2002). However, in aging and neurological populations, movement requires a greater amount of cognitive resources, and when the cognitive resources are exhausted, balance instability and falls may occur (Woollacott and Shumway-Cook, 2002; Al-Yahya et al., 2011; Plummer-D’Amato et al., 2012; Plummer et al., 2013).

Changes in cognitive workload can be observed through changes in pupil size (Sirois and Brisson, 2014). Pupillometry has been used to understand cognitive demand during memory tasks, decision making tasks, and problem solving (Eckstein et al., 2017). The mechanism of pupil dilation due to increased cognitive workload is mediated by a combination of parasympathetic and sympathetic activity. The size of the pupil is controlled by two muscles, the sphincter pupillae and dilator pupillae (Janisse, 1977; Beatty and Lucero-Wagoner, 2000; Larson and Behrends, 2015). The sphincter pupillae is a smooth muscle that is controlled by the parasympathetic fibers of the autonomic nervous system. These parasympathetic fibers originate from the Edinger–Westphal nucleus and are responsible for constricting the pupil (Janisse, 1977; Sirois and Brisson, 2014). The dilator pupillae is also a smooth muscle and is controlled by sympathetic fibers of the autonomic nervous system from the superior sympathetic ganglion, which results in pupil dilation (Janisse, 1977; Sirois and Brisson, 2014). Due to the nature of the innervation of these muscles, changes in pupil size are reflexive (Janisse, 1977; Beatty and Lucero-Wagoner, 2000; Sirois and Brisson, 2014; Larson and Behrends, 2015). With increased attentional or cognitive workload, the locus coeruleus – a small nucleus in the brainstem that regulates arousal, attention, memory, cognitive control, and balance – activates (McGregor and Siegel, 2010). Increased activation of the locus coeruleus subsequently sends inhibitory signals to the Edinger–Westphal nucleus, which leads to pupil dilation by inhibiting parasympathetic fibers (Janisse, 1977; Beatty and Lucero-Wagoner, 2000; Sirois and Brisson, 2014; Larson and Behrends, 2015). Changes in pupil size may therefore indirectly measure locus coeruleus activity resulting from changes in cognitive and postural demand.

Pupillometry is a valid and reliable measure to quantify cognitive workload during cognitive tasks (Eckstein et al., 2017). Studies have shown that pupils dilate with increased task difficulty during various cognitive tasks (Beatty, 1982; Klingner et al., 2011). In addition, pupillometry has been used successfully to examine changes in cognitive workload related to fine motor control reaction time tasks (White and French, 2017). However, pupillometry has not been used in a postural control context. Although postural control requires a small amount of cognitive resources in healthy young adults (Woollacott and Shumway-Cook, 2002), pupillometry has the potential to provide better understanding the cognitive workload of postural control. Thus, pupillometry could be a potential tool to improve physical rehabilitation outcomes through understanding changes in postural demand. The aim of the current study was to examine cognitive workload in healthy young adults during varying postural control and cognitive conditions. We hypothesized that cognitive workload, indexed by pupil size, will increase with a challenging postural control conditions including visual occlusion and cognitive dual tasking.

## Methodology

### Participants

Twenty-one participants between the ages of 18 and 29 were recruited through the University of Kansas Medical Center (*n* = 15) and the University of Illinois at Urbana–Champaign (*n* = 6) in a 2-month time period. Inclusion criteria were self-reported independent ambulation, self-reported normal or corrected-to-normal hearing, self-reported absence of confounding walking or balance impairment, and the ability to speak English. Potential participants were excluded if they had a self-reported history of neurological or vestibular conditions, self-reported presence of musculoskeletal conditions which might affect standing and balance activities, and self-reported complete or partial blindness. All participants were screened for significant cognitive impairment on the Modified Telephone Interview for Cognitive Status (TICS-M); participants who scored below 20 were excluded from the study (de Jager et al., 2003). All recruited participants met the eligibility criteria and were enrolled in the study.

All procedures were approved by the Institutional Review Boards of the University of Kansas Medical Center and the University of Illinois at Urbana–Champaign. Each participant provided written informed consent prior to participation in the study.

### Experimental Design

Upon consenting to take part in the study, participants completed the Montreal Cognitive Assessment (MoCA) (Hobson, 2015). Subsequently, participants were fitted with SMI Remote Eye Tracking Glasses (SensoMotoric Instruments, Teltow, Germany), which recorded pupil size at 60 Hz. The procedures were conducted in a lab space with consistent lighting. Participants performed a series of postural tasks on a Bertec force platform (Bertec, Columbus, OH, United States) at the University of Illinois at Urbana–Champaign or on an AMTI force platform (AMTI OPT464508-1000, Advanced Mechanical Technology, Inc., Watertown, MA, United States) at the University of Kansas Medical Center. At the start of each task, the eye tracking glasses were calibrated using a 3-point calibration according to the manufacturer’s instructions.

**FIGURE 1:**
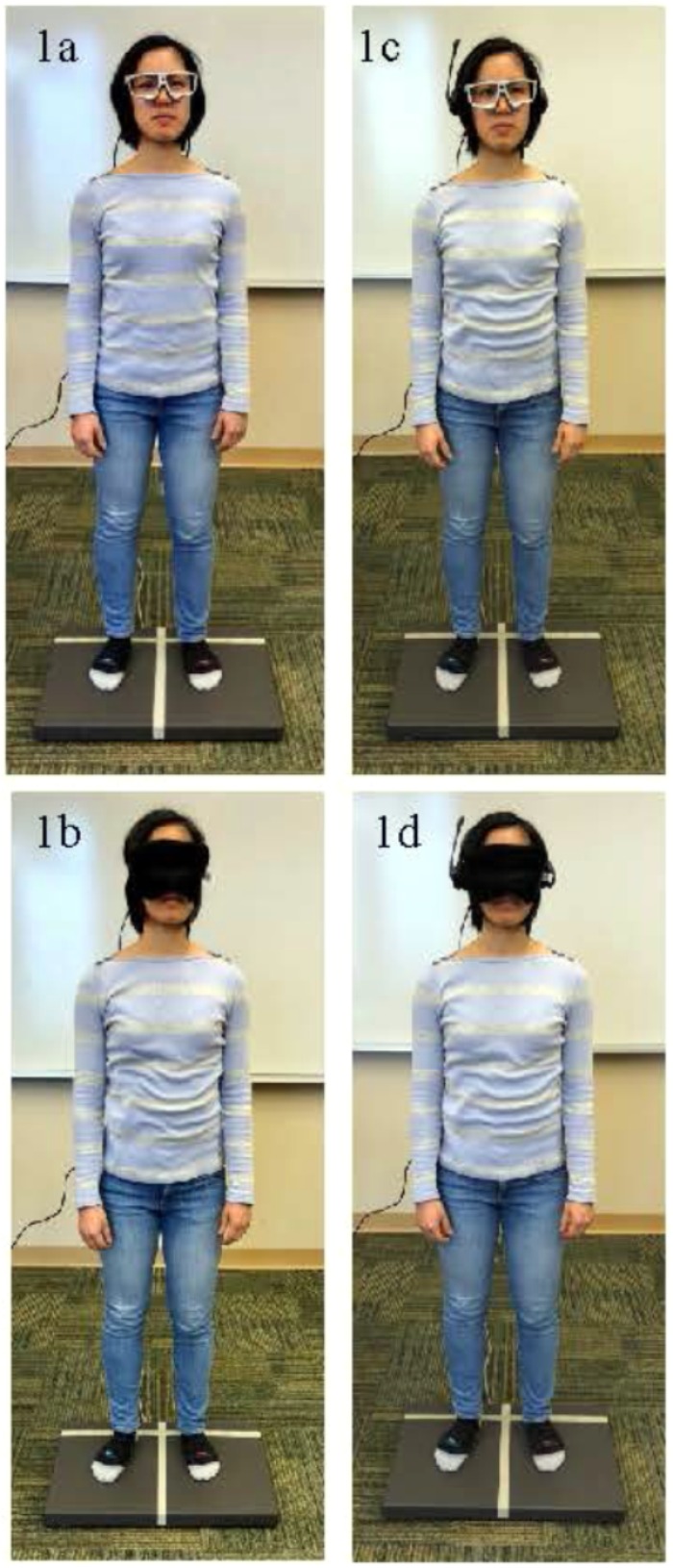
**(a–d)** Depiction of the four postural control conditions. **(a)** Single task standing with eyes open Figure. **(b)** Single task standing with eyes occluded. **(c)** Dual task standing with eyes open. **(d)** Dual task standing with eyes occluded. The person pictured gave consent for publication of these images.

The participants completed four different conditions: (1) single task with eyes open, (2) single task with eyes occluded, (3) dual task with eyes open, and (4) dual task with eyes occluded. **Figure 1** displays the four conditions. In each condition, participants were instructed to look forward and remain as still as possible for 60 s. To ensure participant safety throughout the testing, participants were given a grab bar to stabilize themselves if needed and were fitted with a gait belt. For the first condition, the participants were instructed to focus their eyes on a crosshair target 1.5 m away (**Figure 1a**). For the second condition, after the calibration, the front of the eye tracking glasses was occluded with a sleep mask; participants could not see in front of them, but the eye tracking glasses could still record pupil size (**Figure 1b**). For the third condition, the participants were instructed to focus their eyes on a target 1.5 m away while completing an auditory Stroop task (**Figure 1c**). The auditory Stroop test was shown sensitive to dual task interference in healthy young adults (Kelly et al., 2013). For the auditory Stroop task, participants were instructed to listen to the words “high” and “low.” These words were spoken in a high pitch or a low pitch through headphones. Participants were asked to verbally specify the pitch of the word as quickly as possible (Siu et al., 2008; Plummer-D’Amato et al., 2012). Three different audio files were randomly used for each dual task condition; each audio file contained 15 stimuli with a 2 s interval. Finally, for the fourth task, the eye tracking glasses were occluded with the sleep mask and the auditory Stroop task was performed (**Figure 1d**). During eyes occluded conditions, participants were specifically instructed to keep their eyes open.

The collected eye tracking data were analyzed using SMI BeGaze software (SensoMotoric Instruments, Teltow, Germany) and EyeWorks (EyeTracking Inc., Solana Beach, CA, United States). SMI BeGaze software analyzed the change of the pupil size for each eye throughout the trial. By solely measuring the change of the pupil size, there are potential limitations such as the light reflex interfering with the pupil size and movement artifacts (Marshall, 2007). To combat this potential problem, the EyeWorks software utilized the eye metrics from the SMI BeGaze software to compute the Index of Cognitive Activity (ICA). The ICA is an algorithm that measures cognitive workload through pupil dilation on a continuous scale ranging between 0 (no cognitive workload) and 1 (maximum cognitive workload) (Marshall, 2007). The ICA is computed as the number of unusual increments in pupil size per second. Based on this algorithm the noisy signals such as light reflex are reduced to near zero level (Marshall, 2007). The primary outcome variable was the average ICA value for each eye and for each task. However, in our analysis, we used right eye ICA values to present the results.

The force platforms collected forces Fx, Fy, and Fz and movements Mz, My, and Mz. Center of pressure (CoP) was calculated in the *x* and *y* direction with the following calculations:

$${CoP}_{x}=-My/Fz$$

$${CoP}_{Y}=-Mx/Fz$$

A custom MATLAB code (MathWorks, Natick, MA, United States) employed a 4th order Butterworth filter low pass filter with a cut-off frequency of 10 Hz and resampled the data at 100 Hz. The Bertec force platform collected data at 500 Hz and the AMTI force place collected at 360 Hz. Data were resampled at 100 Hz for consistency between the two force platforms and 100 Hz has been shown to be suitable to characterize CoP variability (Scoppa et al., 2013; Rhea et al., 2015). Average AP and ML CoP sway velocity variables were then calculated for each trial. The secondary outcome variable was the CoP sway velocity in the AP and ML directions as sway velocity has been shown to be a reliable measure of postural stability (Le Clair and Riach, 1996).

### Data Analysis

A two-way ANOVA was run to examine the effect of visual occlusion and cognitive dual tasking on ICA values as well on CoP sway velocity in AP and ML directions. A *post hoc* Sidak test was used to determine the differences in eyes open and eyes occluded conditions. The number of correct responses on the auditory Stroop test was calculated for the dual task conditions.

All variables (except sex) were normally distributed according to Shapiro–Wilk tests. Pearson’s correlation coefficient was used to calculate the relationship between ICA values and CoP sway velocity. The same test was used to analyze the association between the left eye ICA and right eye ICA data. A significance value of 0.05 was used for all significance testing. All the statistical analysis was performed using IBM SPSS Statistics v23.

## Results

**Table 1** summarizes the subjects’ demographic characteristics and the results of global cognitive testing.

**Table 1:** Subject characteristics (*n* = 21).

Characteristics	Mean ± SEM
Age (years)	23.2 ± 0.49
Sex, women, n (%)	12 (57.1)
Education (years)	16.1 ± 0.42
MoCA	28.3 ± 0.35


*Results were reported as mean ± SEM, and as frequency (percentage) for the sex variable. SEM, Standard error of the mean; MoCA, Montreal Cognitive Assessment.*

### Primary Outcome

**FIGURE 2:**
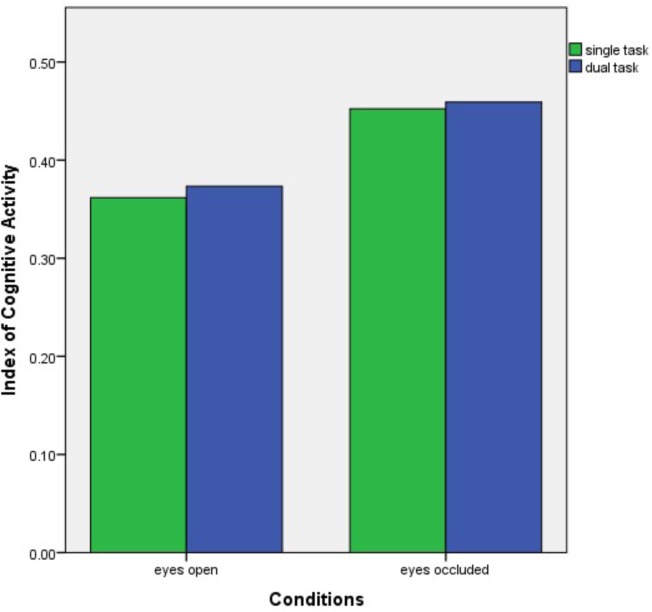
Bar graph of the right eye ICA results over the conditions. ICA, Index of Cognitive Activity.

A two-way ANOVA revealed a significant effect of visual occlusion on ICA values in the right eye (*p* = 0.008), (see **Figure 2**). However, no significant differences were found with the cognitive dual tasking (*p* = 0.77), and no significant interaction was found between the conditions (*p* = 0.94). *Post hoc* analysis demonstrated a significant increase in right eye ICA values from eyes open condition (mean ± standard error mean) (0.36 ± 0.02) to single task eyes occluded condition (0.45 ± 0.02) (*p* = 0.008). No significant effect of condition was observed in the left eye (*p* = 0.15).

We found significant associations between the right eye and left eye ICA results in all four conditions (single task with eyes open *r* = 0.50, *p* = 0.02; single task with eyes occluded *r* = 0.50, *p* = 0.02; dual task with eyes open *r* = 0.40, *p* = 0.05; dual task with eyes occluded *r* = 0.42, *p* = 0.03) suggesting consistency in pupil size recording. However, the results of the left eye were not significant therefore we reported the right eye ICA results.

### Secondary Outcomes

The force platform results demonstrated that there was no significant within-condition effect of visual occlusion as well as cognitive dual tasking, and no significant interaction effect of the conditions on the CoP sway velocity in the AP direction and in the ML direction.

There were no significant differences in incorrect responses of the auditory Stroop test between the dual task eyes open and dual task eyes occluded conditions (*p* = 0.54). The accuracy of responses on the auditory Stroop test was 98.75% ± 3.3 and 97.2% ± 6.2 for the dual task with eyes open and dual task with eyes occluded conditions, respectively. The majority (*n* = 18, 86%) of the subjects completed the auditory Stroop tests without any errors.

### Correlation Analysis Between ICA Values and Force Platform Outcomes

**FIGURE 3:**
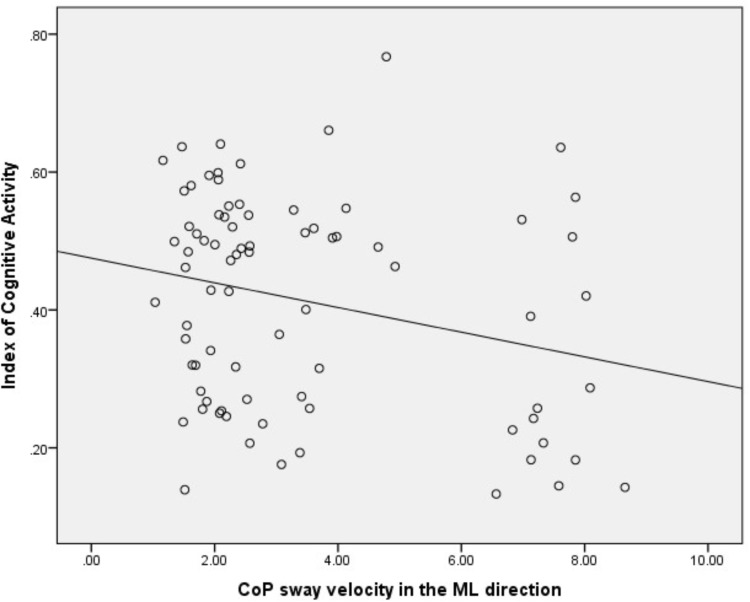
Correlation analysis between ICA and CoP sway velocity in the ML direction. ICA, index of cognitive activity; CoP, center of pressure; ML, medio-lateral.

There was a significant, yet weak, inverse correlation between right eye ICA values and CoP sway velocity in the ML direction across all the conditions (*r* = -0.25, *p* = 0.02) (see **Figure 3**). However, there was no correlation between the right eye ICA values and CoP sway velocity in the AP direction across all the conditions (*r* = -0.17, *p* = 0.13).

## Discussion

The current investigation examined whether challenging postural control through visual occlusion and cognitive dual tasking is associated with increased cognitive workload as measured by pupillometry in healthy young adults. We found that challenging postural demand is associated with greater cognitive workload in healthy young adults. These differences mainly surfaced in postural conditions with visual occlusion. Taken together, these findings suggest that visual occlusion requires additional neural processes in the brain to maintain posture. This increased recruitment of neural processes result in changes in pupil size (increased ICA). However, this phenomenon was not observed when adding a cognitive task to the postural control task in healthy young adults, probably because the cognitive task was not challenging enough.

Several studies have used pupil dilation as an indicator of cognitive workload during cognitive tasks (Eckstein et al., 2017) and motor tasks (Hayashi et al., 2010; Zenon et al., 2014; White and French, 2017). Several studies demonstrated a linear relationship between increased pupil dilation and increased cognitive workload in healthy individuals (Beatty, 1982; Andreassi, 2000; Granholm and Steinhauer, 2004). White and French (2017) demonstrated a positive relationship between increased pupil dilation and increasing motor task difficulty while controlling the mouse to move the cursor over the target from normal to more quick and rapid cursor movements. Another study showed that increased pupil dilation was associated with increased complexity of the physical task (Hayashi et al., 2010). In addition, pupil dilation has been shown to reflect increased effort required to perform a grip task (Zenon et al., 2014). The novelty of the present study is that pupillometry can potentially be used as an indicator of cognitive workload during various challenging postural control tasks in healthy young adults. Using pupillometry might allow researchers to gain insight into the cognitive processes during postural control. Several studies have used other neurophysiological tools to measure cognitive workload during changes in a postural demand in healthy young adults, including functional near infrared spectroscopy (fNIRS) or electroencephalogram (EEG). Herold et al. (2017) demonstrated that healthy young adults had increased frontal brain activation measured by fNIRS during balancing on a balance board. By contrast, Mirelman et al. (2014) did not find changes in frontal brain activation as measured by fNIRS when dual task standing was compared to dual task walking in healthy young adults. Lastly, several EEG studies showed increased activity in the brain during postural balance condition with visual occlusion as well as with cognitive dual tasking both in healthy young and healthy old adults (Ozdemir et al., 2016, 2018). Several reviews discussed the role of cerebral cortex on postural balance and indicated an increase in cognitive workload to maintain postural balance during challenging situations (Jacobs and Horak, 2007; Bolton, 2015). Our results extend the evidence on cerebral activity in postural demanding conditions in healthy young adults. However, compared to the other neurophysiological tools, pupillometry is cost-effective, less intrusive, and easy to implement in clinical practice.

Interestingly, the results showed that CoP sway velocity on the AP and ML directions did not change by visual occlusion or cognitive dual tasking whereas the ICA values significantly increased with increased postural demand by visual occlusion. This might suggest that the behavioral outcomes of postural balance may not be sensitive enough to detect changes in postural demand compared to the neurophysiological response of the brain in healthy young adults. Therefore, pupillometry might help to better understand the cognitive workload related to changes in postural demand in healthy young adults. Furthermore, we found that increased ICA values were significantly correlated with decreased CoP sway velocity in the ML directions. Researchers may need to assess both cognitive workload and force platform data to better understand cognitive and postural adaptations to changes in postural demand.

The lack of effect of the cognitive task on ICA and on COP sway velocity indicates that the Stroop test was not challenging enough evoke higher cognitive workload in healthy young adults. Our results demonstrated that 86% of the individuals from our cohort did not miss any single item from the auditory Stroop test during the dual task conditions. Although several studies reported dual task interference when using the auditory Stroop test (Kelly et al., 2013; Potocanac et al., 2015), some studies demonstrated that this test was not sensitive to observe dual task interference in healthy young adults (Plummer-D’Amato et al., 2012; Tsang et al., 2013). The present study was in line with the latter studies (Plummer-D’Amato et al., 2012; Tsang et al., 2013), therefore we concluded that the auditory Stroop test was not challenging enough to observe dual task interference in healthy young adults. Future studies should take into account task difficulty in order to observe a dual task interference in healthy young adults.

Furthermore, our findings demonstrated that right eye ICA values were more sensitive to demonstrate increased cognitive workload to increased postural demand compared to the left eye. Several studies with animal models and human subjects suggested that pupillary response differs between right and left eyes during increased attentional load possibly due to the brain hemispheric differences (Kim et al., 1998; Liu et al., 2017; Wahn et al., 2017). It is possible that brain hemispheric differences play a role in different responses of the right and left eye in a postural control task. Evidence from a neuroimaging study suggests that left hemisphere is dominant for execution of motor and postural control activities in healthy young adults (Serrien et al., 2006). Although speculative, the increased ICA in the right eye could be explained by increased activation of the left hemisphere due to increased postural demand throughout the testing. However, given the novelty of this result and hypothetical explanation of the mechanism, future studies are needed to investigate the underlying pathways of the hemispheric differences on the pupillary response.

This study has several limitations. The order of the conditions was not randomized for the subjects, which might have resulted in an adaptation to the subsequent condition because of the experience gained in the previous condition. Therefore, the results of this study should be interpreted cautiously. However, the ultimate goal of this research is to examine if pupillometry can be used in older adults and other clinical populations. Clinical assessment of postural control in clinical populations involves progressively difficult balance tasks to maximize participant safety. Nevertheless, to minimize this adaptation, we gave breaks between the conditions and used different auditory Stroop tests for the dual task conditions. In addition, although we standardized the ambient lighting while testing the subjects, the ambient lighting might have been different between the two testing sites. However, in this study, we used the ICA algorithm to filter out the noise of ambient lighting (Marshall, 2007). Therefore, the combined results from the two sites truly reflect increased pupil size due to increased cognitive workload. In addition, the selection of the cognitive task (auditory Stroop) was not successful in eliciting either increased cognitive workload or augmented CoP sway velocity in healthy young adults. This might suggest that cognitive task manipulation was not effective to evoke behavioral or neurophysiological changes. Future studies should consider effective cognitive task manipulation to observe dual task interference in healthy young adults. Nevertheless, this study contributes to the current literature to utilize pupillometry in the design of postural control studies. Lastly, although we observed increased cognitive workload with visual occlusion during quiet standing, we did not capture activated areas of the brain during the conditions. A more robust design would be a combined approach in which EEG or fNIRS is used with pupillometry. Overall, this study will build a knowledge to implement pupillometry to assess cognitive workload during increased postural demand in older adults with and without neurological conditions.

## Conclusion

The present study provides support for cognitive workload changes measured by pupillometry related to changes in postural control in healthy young adults. Through increasing postural demand by visual occlusion, a greater pupil size (ICA) was observed possibly due to increased neural processing in the cerebral cortex to maintain posture. Future studies with similar experimental design are needed in healthy older individuals and those with neurological conditions to assess differences in cognitive workload related to aging and disease during challenging postural control tasks.

## Author Contributions

MK, TW, JS, and HD conceptualizing the study. MK and TW drafting the manuscript. MK data analysis. HD and JS valuable suggestions and manuscript review.

## Conflict of Interest Statement

The authors declare that the research was conducted in the absence of any commercial or financial relationships that could be construed as a potential conflict of interest.
